# High prevalence of binge drinking among people living with HIV in four African countries

**DOI:** 10.1002/jia2.25202

**Published:** 2018-12-14

**Authors:** Marcellin N Nouaman, Michael Vinikoor, Moussa Seydi, Didier K Ekouevi, Patrick A Coffie, Lloyd Mulenga, Aristophane Tanon, Matthias Egger, François Dabis, Antoine Jaquet, Gilles Wandeler, IeDEA

**Affiliations:** ^1^ Programme PACCI CHU de Treichville Abidjan Côte d'Ivoire; ^2^ Department of Medicine University of Alabama at Birmingham Birmingham AL USA; ^3^ Centre for Infectious Disease Research in Zambia (CIDRZ) Lusaka Zambia; ^4^ Service de maladies infectieuses et tropicales CRCF, CHU de Fann Dakar Sénégal; ^5^ INSERM U1219 Bordeaux Population Health Research ISPED Université de Bordeaux Bordeaux France; ^6^ Département de santé publique Faculté des Sciences de la santé Université de Lomé Lomé Togo; ^7^ CHU de Treichville Service de maladies infectieuses et tropicales Abidjan Côte d'Ivoire; ^8^ University Teaching Hospital Lusaka Zambia; ^9^ Zambia Ministry of Health Lusaka Zambia; ^10^ Institute of Social and Preventive Medicine University of Bern Bern Switzerland; ^11^ Centre for Infectious Diseases Epidemiology and Research University of Cape Town Cape Town South Africa; ^12^ Department of Infectious Diseases Bern University Hospital University of Bern Bern Switzerland

**Keywords:** alcohol, binge drinking, HIV, antiretroviral therapy, viral hepatitis, sub‐Saharan Africa

## Abstract

**Introduction:**

Excessive alcohol consumption leads to unfavourable outcomes in people living with HIV (PLHIV), including reduced adherence to antiretroviral therapy (ART) and engagement into care. However, there is limited information on alcohol consumption patterns among PLHIV in sub‐Saharan Africa.

**Methods:**

Using a cross‐sectional approach, the Alcohol Use Disorders Identification Test (AUDIT‐C) was administered to PLHIV attending HIV clinics in Côte d'Ivoire, Togo, Senegal and Zambia (2013 to 2015). Hazardous drinking was defined as an AUDIT‐C score ≥4 for men or ≥3 for women, and binge drinking as ≥6 drinks at least once per month. The prevalence of binge drinking was compared to estimates from the general population using data from the World Health Organization. Factors associated with binge drinking among persons declaring any alcohol use in the past year were assessed using a logistic regression model to estimate odds ratio (OR) and their corresponding 95% confidence intervals (CI).

**Results:**

Among 1824 PLHIV (median age 39 years, 62.8% female), the prevalence of hazardous alcohol use ranged from 0.9% in Senegal to 38.4% in Zambia. The prevalence of binge drinking ranged from 14.3% among drinkers in Senegal to 81.8% in Zambia, with higher estimates among PLHIV than in the general population. Male sex (OR 2.4, 95% CI 1.6 to 3.7), tobacco use (OR 1.7, 95% CI 1.0 to 2.9) and living in Zambia were associated with binge drinking.

**Conclusions:**

Alcohol consumption patterns varied widely across settings and binge drinking was more frequent in HIV‐positive individuals compared to the general population. Interventions to reduce excessive alcohol use are urgently needed to optimize adherence in the era of universal ART.

## 1 Introduction

Sub‐Saharan Africa (SSA) has the highest prevalence of HIV globally, and accounts for 68% of people living with HIV (PLHIV) 1. Many countries in the region have high prevalence estimates of hazardous alcohol consumption, which has been shown to be higher among PLHIV compared to the general population 2. Importantly, heavy episodic drinking, or “binge drinking,” is common among young adults 3, 4. Specific determinants of alcohol consumption vary widely across settings as they are driven by a multitude of cultural, social, financial and religious determinants 5. As assessing and addressing alcohol consumption have not been priorities in routine HIV care in many clinical settings, knowledge about this important comorbidity remains limited.

Excessive alcohol consumption leads to increased risk behaviour, notably condomless and coercive sex, which increase levels of HIV transmission 6, 7, 8. Furthermore, it also has negative implications for the health of PLHIV: it has been associated with low rates of antiretroviral therapy (ART) initiation, poor engagement in medical care, virological failure and reduced survival 9, 10, 11. The negative impact of alcohol consumption on ART adherence mediates many of these complications. In a study among US veterans, a dose–response relationship between alcohol consumption and adherence was found: periods of lower adherence often followed episodes of alcohol consumption, and adherence decreased as levels of alcohol consumption increased 12. In SSA, alcohol consumption has been recognized as one of the most important causes of non‐adherence to ART, especially in men 13. Recent data from a large randomized trial conducted in low‐ and middle‐income countries showed that binge drinking had a strong impact on ART adherence, especially on weekends 14. Importantly, living with HIV has been associated with additional risk factors of liver and cardiovascular disease in SSA, including viral hepatitis B and C infections, and tobacco use. A recent meta‐analysis found a higher level of tobacco use among HIV‐positive persons from low‐ and middle‐income countries compared to negative ones 15. However, there is limited information on how HIV, tobacco and alcohol use intersect in SSA.

Despite the recognized negative impact of excessive alcohol consumption on the health status of PLHIV, the prevalence and patterns of alcohol use among HIV‐positive individuals attending clinical care in SSA have not been widely described. We aimed at evaluating alcohol consumption patterns among PLHIV from four countries in West and Southern Africa. Furthermore, we compared the prevalence of binge drinking among the PLHIV participating in our study with estimates for the corresponding general population.

## 2 Methods

### 2.1 Study context and data sources

We performed a cross‐sectional study of alcohol consumption among HIV‐positive patients attending care in four large urban HIV clinics. In West Africa, 1050 PLHIV were enrolled at referral hospitals in Abidjan, Côte D'Ivoire (n = 350), Lomé, Togo (n = 350) and Dakar, Senegal (n = 350) between 2013 and 2015 16. In each clinic, a total of 10 to 15 patients were randomly selected each day, during a period of three months. In Lusaka, Zambia, 798 consecutive PLHIV were included at the time of ART initiation between 2013 and 2014 17. All sites were part of the International epidemiologic Databases to Evaluate AIDS (IeDEA) network, a large international cohort collaboration 18. Data from Zambia, Senegal and Togo were cleaned and merged with the existing database from Abidjan, Côte d'Ivoire. The study was approved by the National Ethics Committees from Côte d'Ivoire (no. 036/MSLS/CNER‐dkn), Togo (no. 117/MJRIR/SG/DAPR), Senegal (no. 0178/MSAS/DPRS/CNERS), and by the ethics committees of the University of Zambia (Lusaka, Zambia) and University of Alabama at Birmingham (Birmingham, USA) for the Zambian site. All patients signed an informed consent to participate in the study.

### 2.2 Study measurements

Using a common protocol, we collected information on the following demographic, clinical and behavioural characteristics: age, sex, marital status, education level, CD4 cell count, body mass index, transaminases and ART history. Tobacco consumption was reported as “never smoker” or “current or past smoker.” Viral hepatitis infections were assessed using rapid diagnostic tests: Determine^®^ (Alere, Waltham, MA, USA) for hepatitis B surface antigen (HBsAg) and Oraquick^®^ (Orasure, Bethlehem, PA, USA) for anti‐hepatitis C virus (HCV) antibodies, as these tests have shown relatively good diagnostic accuracy, including a validation study performed at our Zambian study site 19, 20.

### 2.3 Assessment of alcohol consumption

In all sites, we assessed alcohol consumption using the abbreviated version of the Alcohol Use Disorders Identification Test (AUDIT‐C) questionnaire, which consists of three questions focusing on the quantity and frequency of alcohol consumption 21. Nurses specifically trained for this purpose administered the questionnaires. The three questions have five possible answers, each of them giving between 0 and 4 points. Thus, the maximum score is 12: score = 0 defines non‐drinkers; a score between 1 and 3 defines moderate drinkers; and a score above 3 (women) or 4 (men) defines hazardous drinking. Binge drinking was defined based on the third question of the AUDIT‐C (≥6 drinks on one occasion at least monthly). Our research associates were trained to convert alcoholic beverages consumed on a typical day in number of standard drinks. Examples were described on leaflets presenting the different kinds of drinks (size and types) and what was considered as a standard unit of alcohol intake (glass of wine of 14 cL, glass of beer of 25 cL, glass of gin or locally brewed palm wine of 4 cL, etc.). In order to contextualize binge drinking estimates from our study population, we also describe estimates from the general population using data from the Global Status Report on Alcohol and Health 2014 from the World Health Organization (WHO) 4. These are based on a wide range of sources in each country, including published surveys, previous WHO‐led surveillance studies and alcohol sales data. According to WHO, a standard drink contains 13.5 g of alcohol. Binge drinking or heavy episodic drinking would be defined as 60 or more grams of pure alcohol (on at least one single occasion at least monthly), which is slightly lower than the AUDIT‐C‐derived cut‐off used to define binge drinking in our study.

### 2.4 Statistical analyses

We compared patient demographics and clinical characteristics between countries using Student's *t*‐test, the non‐parametric Mann–Whitney *U*‐test or analysis of variance for continuous variables. We used the Pearson's chi‐square test or the Fisher's exact test to compare frequencies. An unconditional logistic model was used to identify factors associated with binge drinking among participants reporting any alcohol use during the past 12 months. Variables statistically associated with binge drinking (*p* < 0.20) in univariable analyses were retained and introduced into the initial multivariable model. A backward stepwise regression procedure was applied to select the final multivariate model. Adjusted odds ratios (aOR) were estimated with their 95% confidence intervals (CI). A *p* < 0.05 was considered for statistical significance in the final model. In sensitivity analyses, we evaluated the proportion of the whole study population that reported binge drinking in each clinic, and repeated the multivariable analyses in the full study population. All analyses were performed with Stata software (StataTM 12.0, College Station, TX, USA).

## 3 Results

### 3.1 General characteristics

Of 1850 patients included in the database, 23 from West Africa and three from Zambia had missing AUDIT‐C scores and were excluded from the analyses. Data from 795 PLHIV from Zambia and 1029 from West Africa were analysed. The median age of the participants was 34 years (interquartile range [IQR] 29 to 40) in Zambia versus 43 years (IQR 37 to 50) in West Africa (Table 1). In Zambia, women accounted for 53.7% of patients whereas 70.0% of the subjects were women in West Africa. Participants from Senegal were less likely to have access to schools (41.2%) compared to participants from other countries. The proportion of patients reporting present or past tobacco consumption was relatively high in Senegal (26.5%) and Côte d'Ivoire 61 (17.5%). In Togo, only 51.1% of PLHIV were on ART, whereas this was the case for 96% of them in Côte d'Ivoire and Senegal, and 100% in Zambia. The proportion of PLHIV with initial CD4 counts >200 cells/mm^3^ was highest in Zambia (54.6%) and lowest in Senegal (31.0%). Overall, the prevalence of positive anti‐HCV antibody was 0.8% (95% CI 0.4 to 1.2), and 10.6% (95% CI 9.2 to 12.1) had a positive HBsAg test.

**Table 1:** General characteristics of HIV‐positive patients, by country

	Côte d'Ivoire n = 349	Senegal n = 328	Togo n = 352	Zambia n = 795	*p*
Male sex (%)	98 (28.1)	108 (32.9)	104 (29.5)	368 (46.3)	<0.001
Marital status (%)					
Never married	154 (44.1)	94 (28.7)	103 (29.3)	75 (9.5)	<0.001
Divorced	89 (25.5)	1 (0.3)	2 (0.6)	145 (18.4)	
Married	63 (18.1)	152 (46.3)	193 (55.0)	487 (61.9)	
Widowed	43 (12.3)	81 (24.7)	53 (15.1)	80 (10.2)	
Median age in years (IQR)	43 (38 to 50)	45 (39 to 53)	40 (33 to 48)	34 (29 to 40)	0.001
Education level (%)					
None	74 (21.2)	135 (41.2)	53 (15.3)	46 (5.8)	<0.001
Primary	71 (20.3)	79 (24.0)	110 (31.8)	136 (17.3)	
Secondary	153 (43.8)	80 (24.4)	149 (43.1)	583 (74.2)	
University	51 (14.6)	34 (10.4)	34 (9.8)	21 (2.7)	
Median BMI (IQR)	23.5 (21 to 26.4)	21.5 (18.7 to 25)	22.7 (20.3 to 25.3)	20.3 (18.3 to 22.7)	0.001
Median CD4 count in cells/μL (IQR)	219 (82 to 352)	123 (47 to 234)	193 (91 to 296)	228 (118 to 337)	0.001
Tobacco consumption (%)					
Never smokers	288 (82.5)	241 (73.5)	321 (91.7)	709 (89.2)	<0.001
Present/past history of smoking	61 (17.5)	87 (26.5)	29 (8.3)	86 (10.8)	
Positive HBs antigen (%)	35 (10.0)	40 (12.2)	21 (6.0)	98 (12.3)	<0.001
Positive anti‐HCV antibody (%)	1 (0.3)	6 (1.8)	7 (2.0)	1 (0.1)	<0.001

BMI, body mass index; HCV, hepatitis C virus; IQR, interquartile range.

### 3.2 Alcohol consumption among HIV‐positive individuals

**Figure 1:**
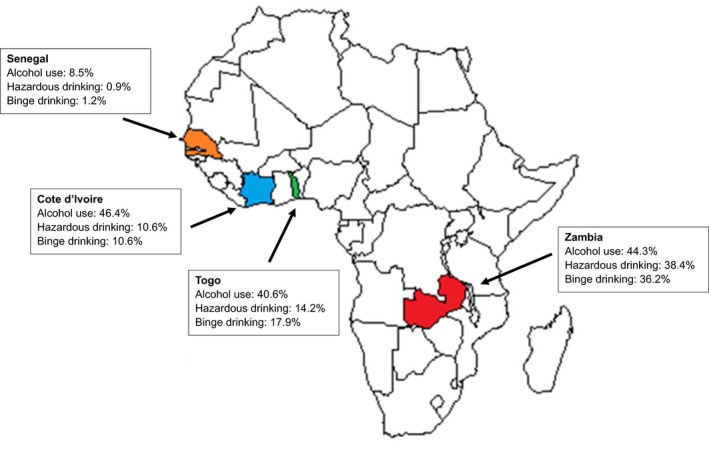
Prevalence of reported current alcohol consumption, hazardous drinking and binge drinking in the past year among people living with HIV in three clinics in West Africa and one in Southern Africa, according to the Alcohol Use Disorders Identification Test definition

**Figure 2:**
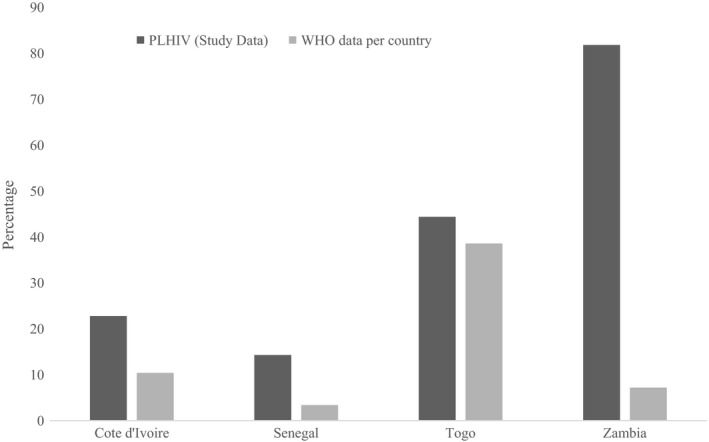
Prevalence of binge drinking among persons declaring any alcohol use in HIV‐positive individuals and the general population, by country

Table 2 and Figure 1 show self‐reported alcohol consumption among 1824 HIV‐positive patients across the four clinical sites. A total of 685 participants (37.5%, 95% CI 35.3 to 39.8) reported any alcohol consumption in the past year and the overall prevalence of hazardous alcohol consumption was 21.7% (95% CI 19.8 to 23.5). Patients from Senegal were more likely to report alcohol abstinence (91.5%), compared to participants from other countries, where levels of abstinence ranged from 53.6% in Côte d'Ivoire to 59.4% in Togo. The proportion of patients reporting hazardous alcohol consumption was higher in Zambia (38.4%) than in Togo (14.2%), Côte d'Ivoire (10.6%) and Senegal (0.9%).The overall prevalence of binge drinking was 1.2% in Senegal, 10.6% in Côte d'Ivoire, 17.9% in Togo and 36.2% in Zambia. Among patients reporting any alcohol use in the past year, the estimated prevalence of binge drinking ranged from 14.3% in Senegal to 81.8% in Zambia. In all countries surveyed, hazardous alcohol consumption and binge drinking were more frequent among men compared to women (Table 2). Figure 2 describes estimates of binge drinking obtained in our study and in the general population of each country.

**Table 2:** Prevalence of alcohol use, hazardous drinking and binge drinking according to participating country and gender

	Côte d'Ivoire n = 349	Senegal n = 328	Togo n = 352	Zambia n = 795	Total n = 1824	*p*
AUDIT‐C score: overall (%)						
0 (non‐drinker)	187 (53.6)	300 (91.5)	209 (59.4)	443 (55.7)	1139 (62.4)	<0.001
1 to 3 (moderate drinker)	125 (35.8)	25 (7.6)	93 (26.4)	47 (5.9)	290 (15.9)	
≥4 (hazardous drinker)	37 (10.6)	3 (0.9)	50 (14.2)	305 (38.4)	395 (21.7)	
Binge drinking overall (%)^a^	37 (10.6)	4 (1.2)	63 (17.9)	288 (36.2)	392 (21.5)	<0.001
Binge drinking among drinkers (%)^a^	37 (22.8)	4 (14.3)	63 (44.1)	288 (81.8)	392 (57.2)	<0.001
AUDIT‐C score: men (%)						
0 (non‐drinker)	49 (50.0)	94 (87.0)	43 (41.4)	140 (38.1)	326 (48.1)	<0.001
1 to 3 (moderate drinker)	27 (27.5)	11 (10.2)	29 (27.9)	13 (3.5)	80 (11.8)	
≥4 (hazardous drinker)	22 (22.5)	3 (2.8)	32 (30.7)	215 (58.4)	272 (40.1)	
Binge drinking overall (%)^a^	21 (21.4)	2 (1.8)	37 (35.6)	196 (53.3)	256 (37.8)	<0.001
Binge drinking among drinkers (%)^a^	21 (42.9)	2 (14.3)	37 (60.7)	196 (85.9)	256 (72.7)	<0.001
AUDIT‐C score: women (%)						
0 (non‐drinker)	138 (55.0)	206 (93.6)	166 (66.9)	303 (71.0)	813 (71.0)	<0.001
1 to 2 (moderate drinker)	93 (37.1)	13 (5.9)	58 (23.4)	19 (4.4)	183 (16.0)	
≥3 (hazardous drinker)	20 (7.9)	1 (0.5)	24 (9.7)	105 (24.6)	150 (13.0)	
Binge drinking overall (%)^a^	16 (6.4)	2 (0.9)	26 (10.5)	92 (21.6)	136 (11.9)	<0.001
Binge drinking among drinkers (%)^a^	16 (14.2)	2 (14.3)	26 (31.7)	92 (74.2)	136 (40.8)	<0.001

a Binge drinking: ≥6 drinks on one occasion at least monthly (based on question no. 3 of AUDIT‐C questionnaire).

### 3.3 Risk factors of binge drinking

In multivariable analyses, male sex (aOR 2.4, 95% CI 1.6 to 3.7) and using tobacco (aOR 1.7, 95% CI 1.0 to 2.9) were associated with binge drinking among patients having reported any drinking (Table 3). Patients from Zambia were much more likely to binge drink compared to those of other countries. In analyses, including the full study population in each clinic, living in Zambia, male sex and tobacco use remained significant risk factors for binge drinking. In addition, age <35 years, nadir CD4 count >200 cells/μL and HBsAg‐positivity were significant risk factors for binge drinking in the model including drinkers as well as non‐drinkers (Table S1).

**Table 3:** Factors associated with binge drinking among HIV‐positive patients reporting any alcohol use during the past 12 months (n = 684)

	Univariable analyses			Multivariable analyses	
	n/N	OR (95% CI)	*p*	OR (95% CI)	*p*
Study site					
Lusaka, Zambia	288/352	1	<0.001	1	0.001
Abidjan, Côte d'Ivoire	37/162	0.07 (0.04 to 0.1)		0.07 (0.04 to 0.1)	
Dakar, Senegal	4/28	0.04 (0.01 to 0.1)		0.03 (0.01 to 0.08)	
Lomé, Togo	63/143	0.2 (0.1 to 0.3)		0.2 (0.1 to 0.3)	
Sex					
Female	136/333	1	<0.001	1	0.001
Male	256/352	4.0 (2.8 to 5.6)		2.4 (1.6 to 3.7)	
Age in years					
≥35	216/426	1	<0.001	1	0.9
<35	176/259	2.1 (1.5 to 2.8)		0.9 (0.6 to 1.5)	
Marital status (n = 682)					
Never married	68/167	1	0.002		
Divorced	64/105	2.3 (1.4 to 3.7)			
Married	230/338	3.1 (2.1 to 4.5)			
Widowed	28/72	0.9 (0.5 to 1.6)			
Education level (n = 680)					
None	25/58	1	0.001		
Primary	67/132	1.4 (0.7 to 2.5)			
Secondary	268/422	2.3 (1.3 to 4.0)			
University	29/68	0.9 (0.5 to 1.9)			
Tobacco consumption					
Never smokers	291/532	1	0.01	1	0.03
Present/past history of smoking	101/152	1.6 (1.1 to 2.4)		1.7 (1.04 to 2.9)	
HBs antigen					
Negative	337/600	1	0.13		
Positive	55/85	1.4 (0.9 to 2.3)			
Nadir CD4 count in cells/μL					
≤200	39/135	1	<0.001		
>200	353/550	4.4 (2.9 to 6.6)			

## 4 Discussion

Across four large urban HIV clinics in West and Southern Africa, hazardous alcohol consumption was reported by 13% of women and 40% of men, whereas among drinkers, 41% of women and 73% of men described episodes of binge drinking. Compared with the general population of each country, the prevalence of binge drinking was much higher in HIV‐positive individuals. Considering the impact of excessive alcohol consumption on ART adherence and engagement into care, our results highlight the need for a better understanding of the determinants and risk factors of alcohol use for evaluating interventions to reduce alcohol consumption among HIV‐positive populations in SSA.

The systematic collection of data on alcohol consumption patterns using the AUDIT‐C score, a validated tool previously used in African settings, among close to 2000 HIV‐positive patients allowed us to consider our robust estimates in the light of published estimates from the general population of each country. Among participants who reported any alcohol consumption in the previous 12 months, 82% in Lusaka and 44% in Lomé reported episodes of binge drinking. In the four countries surveyed, we found a much higher prevalence of binge drinking in HIV‐positive patients compared to WHO estimates among uninfected populations. The largest difference between populations was observed in Zambia, where 81.1% of PLHIV but only 7.2% of the general population reported binge drinking. In Côte d'Ivoire (22.8% in PLHIV and 10.4% in the general population) and Senegal (14.3% in PLHIV and 3.4% in the general population), differences in prevalence of binge drinking were moderate, whereas estimates from both populations were more similar in Togo (44.1% in PLHIV and 38.6% in the general population). Several recent studies described high alcohol consumption levels among HIV‐positive individuals in SSA 22. For instance, in a study of >3000 individuals recruited at inpatient and outpatients clinics in Kampala, Uganda, individuals living with HIV were nearly twice as likely to report current hazardous drinking as HIV‐uninfected participants 23. Reasons for the increased alcohol consumption among urban HIV‐positive populations in SSA include HIV‐associated stigma, stress and depression, precarious socio‐economic situations and unemployment 24.

We found large differences in alcohol consumption patterns across countries, potentially driven by contextual differences. Hazardous alcohol consumption affected over 10% of the study population in Zambia, Côte d'Ivoire and Togo, but was uncommon in Senegal (0.9% of participants), where the large majority of the population is Muslim. We previously found similar estimates in a study among 680 prison inmates in Senegal and Togo: 12% of prisoners in Lomé reported hazardous drinking versus 5% in Senegal 25. Similar differences in alcohol consumption patterns were found in Tanzania: alcohol consumption was less common in adolescents of Muslim faith compared to others, independent of the region where the survey was conducted 26.

In all cohorts included in our study, men were more likely to show excessive alcohol consumption compared to women. Among men who reported any alcohol consumption in the previous 12 months, 86% in Lusaka and 61% in Lomé reported episodes of binge drinking. In analyses adjusted for common confounders, men were three times more likely to report binge drinking compared to women. These results are consistent with findings from high‐income countries, where binge drinking, an increasing public health problem among adolescents, is known to be more common among men than women 27, 28. In these settings, long‐term follow‐up studies have shown that binge‐drinking male adolescents frequently continue to do so and have a high risk of chronic alcohol consumption in adulthood 29. One exception in our study was the group of female participants from Zambia, who also showed extremely high levels of binge drinking (74%). However, the younger age of participants from Zambia might have in part explained this result. Although the long‐term consequences of specific drinking patterns have not been thoroughly assessed in individuals living with HIV in SSA, the situation in Zambia, and, to a lesser extent in West Africa, is worrisome and warrants dedicated interventions to limit the detrimental effects of alcohol in these populations.

This is one of the first large studies focusing on the detailed assessment of alcohol consumption patterns among HIV‐positive individuals in SSA. The standardized evaluation of drinking patterns as well as the collection of many potential risk factors using the same methodology across all sites allowed us to make sound comparisons between countries. Unfortunately, WHO does not express alcohol consumption based on AUDIT‐C criteria, but rather as the number of litres of pure alcohol per year. Therefore, we were not able to compare hazardous drinking between HIV‐positive and uninfected persons and the measures for binge drinking, although comparable, were not identical. Although we gathered data from various geographical areas, our study population, which we enrolled at urban tertiary care clinics, was not representative of the HIV‐positive population in each country. Furthermore, the study was restricted to three West African countries and one cohort in Southern Africa, therefore limiting our ability to extrapolate our findings to other countries in SSA. The self‐reported nature of alcohol consumption is another limitation of our study, as it may have led to reporting bias, especially in settings where drinking is not socially acceptable. Thus, our estimates as well as those from WHO, may have underestimated the prevalence of excessive alcohol consumption, especially in settings such as Muslim countries, where social desirability might have played a role. Furthermore, it can be difficult to quantify alcohol use in African settings where people drink traditional alcoholic beverages with variable alcohol content, and use nontraditional containers. Finally, the cross‐sectional design of our study did not allow the investigation of factors associated with changing dynamics and patterns of alcohol consumption.

## 5 Conclusions

Our study showed a very high prevalence of excessive alcohol consumption among HIV‐positive populations in four African countries, and highlights the diversity in drinking patterns across settings and subpopulations. Considering the dramatic impact of alcohol use disorder on health‐related issues but also on families and society as a whole, it is urgent to consider this issue as a public health threat in SSA. Hopefully, the results of our study will help inform the design and implementation of tailored interventions to prevent and/or reduce alcohol consumption among PLHIV in Africa. Behavioural interventions focusing on alcohol use reduction showed positive results in PLHIV 30. However, interventions such as motivational interviewing have been poorly studied in SSA to date and deserve more attention 31. Considering the potential impact of excessive alcohol consumption on adherence to ART and HIV care, addressing this issue might prove useful for the success of the “treat all” ART strategy which is being rolled‐out across SSA.

## Competing interests

All authors declare that they have no competing interests.

## Authors’ contributions

MN, MV, AJ and GW designed the study, contributed to data analyses and wrote the first draft of the manuscript. MN, AJ, GW, MV, AT, PC, DE, LM, MS, ME and FD all contributed to interpretation of data, critically reviewed the manuscript and agreed on its final version.

## Supporting information


**Table S1.** Factors associated with binge drinking among the full study population of HIV‐positive patients (n = 1824)
