# Supplementary material for: High prevalence of binge drinking among people living with HIV in four African countries
Source: J Int AIDS Soc. 2018 Dec 14;21(12):e25202. doi: 10.1002/jia2.25202 (PMC6294116; doi:10.1002/jia2.25202)
Supplement: Supplementary file 1 — Table S1. Factors associated with binge drinking among the full study population of HIV‐positive patients (n = 1824) [file JIA2-21-e25202-s001.docx]

**Supplementary Table 1: Factors associated with binge drinking among the full study population of HIV-infected patients (n=1824)**

|  | Univariable analysis | | | Multivariable analysis | |
| --- | --- | --- | --- | --- | --- |
|  | **n/N** | **OR (95% CI)** | **p** | **OR (95% CI)** | **P** |
| Study site |  |  | <0.001 |  | <0.001 |
| Lusaka, Zambia | 305/795 | 1 |  |  |  |
| Abidjan, Côte d’Ivoire | 37/349 | 0.2 (0.1 – 0.3) |  | 0.2 (0.1 – 0.4) |  |
| Dakar, Senegal | 3/328 | 0.01 (0.005 – 0.05) |  | 0.01 (0.003 – 0.04) |  |
| Lomé, Togo | 50/352 | 0.3 (0.2 – 0.4) |  | 0.4 (0.3 – 0.7) |  |
| Sex |  |  | <0.001 |  | <0.001 |
| Female | 123/1146 | 1 |  | 1 |  |
| Male | 272/678 | 5.9 (4.5 – 7.1) |  | 4.3 (3.2 – 5.5) |  |
| Age in years |  |  | <0.001 |  | 0.03 |
| ≥35 | 206/1209 | 1 |  | 1 |  |
| <35 | 186/615 | 2.1 (1.6 – 2.6 ) |  | 1.4 (1.1 - 1.8) |  |
| Marital status (n=682) |  |  | <0.001 |  |  |
| Never married | 69/426 | 1 |  |  |  |
| Divorced | 67/237 | 2.0 (1.4 – 2.9) |  |  |  |
| Married | 231/895 | 1.8 (1.3– 2.4) |  |  |  |
| Widowed | 26/257 | 0.6 (0.4 – 0.9) |  |  |  |
| Education level (n=680) |  |  | <0.001 |  |  |
| None | 26/308 | 1 |  |  |  |
| Primary | 56/396 | 1.8 (1.1 – 2.9) |  |  |  |
| Secondary | 280/965 | 4.4 (2.9 – 6.8) |  |  |  |
| University | 28/140 | 2.7 (1.6 – 4.8) |  |  |  |
| Tobacco consumption |  |  | <0.001 |  | <0.001 |
| Never smokers | 286/1559 | 1 |  | 1 |  |
| Present/past history of smoking | 109/263 | 3.1 (2.4 – 4.1) |  | 5.2 (3.4 – 7.9) |  |
| HBs antigen |  |  | 0.004 |  | 0.04 |
| Negative | 337/1630 | 1 |  | 1 |  |
| Positive | 58/194 | 1.6 (1.1 – 2.3) |  | 1.5(1.02 – 2.3) |  |
| Nadir CD4 count in cells/µL |  |  | <0.001 |  |  |
| ≤200 | 30/510 | 1 |  | 1 | 0.001 |
| >200 | 365/1314 | 6.2 (4.2 – 9.1) |  | 2.4 (1.4 – 3.9) |  |
